# Hepatitis B Vaccination: Needs a Revision

**DOI:** 10.5812/hepatmon.17461

**Published:** 2014-03-11

**Authors:** Mohammad Saeid Rezaee-Zavareh, Behzad Einollahi

**Affiliations:** 1 Students’ Research Committee, Baqiyatallah University of Medical Sciences, Tehran, IR Iran; 2 Middle East Liver Disease Center, Tehran, IR Iran; 3 Nephrology and Urology Research Center, Baqiyatallah University of Medical Sciences, Tehran, IR Iran

**Keywords:** Hepatitis B Antibodies, Hepatitis B Vaccines, Vaccination, Hepatitis B Surface Antigens

## 1. Hepatitis B virus Infection, Epidemiology and Complications

It is common knowledge that hepatitis B virus (HBV) infection is an important worldwide health issue which can lead to acute and chronic infection Fulminant hepatitis may be the result of the acute infection. HBV infection in chronic condition can also increase the risk of some more important complications such as hepatic decompensation, cirrhosis and even eventually hepatocellular carcinoma (HCC) (1, 2). Based on the world health organization (WHO) reports, there are more than two billion individuals infected with HBV infection around the world (3). Almost 350 million people have chronic liver disease and the mortality rate, as a consequence of hepatitis B is approximately one million each year as well as over 4 million new cases of HBV infections (3).

## 2. History of HBV Vaccine and Its Generations

Like for many other infectious diseases, vaccination, as a preventive method, plays an important role in disease control of the HBV infection (4). Until now, three generations of HBV vaccines has been introduced. Krugman’s observation about immunogenicity of HBsAg and the protective effects of anti-HBS antibody against HBV finally led to generating the first vaccine generation (1) containing inactivated HBsAg driven from the human carrier plasma of the HBV infected individuals. This vaccine was produced by Merck and at the same time by the Pasteur institute. Subsequently, it was approved by the FDA in 1981 (1). Recombinant DNA technology let to making second generation of HBV vaccines by using the yeast Saccharomyces cerevisiae with these two formulas; Engerix B and Recombivax HB. These types of vaccination also contain HBsAg. However, third generation vaccines are considerably more immunogenic, than HBsAg due to their uses of pre-S1 and pre-S2 antigens, but they aren’t used widespread yet. Third generation vaccines are also manufactured with recombinant DNA technology by mammalian cells (1, 5). 

After the WHO suggestion in 1991 for application of a widespread HBV vaccination by 1997, many countries including the USA and some regions of East Asia and Europe saw a significant decrease in prevalence of the HBV infection (5, 6), childhood HCC and fulminant hepatitis. (6). Nowadays, HBV vaccines, routinely the second generation, are administered three times (on 0, 1 and 6 months period) by intramuscular (deltoid) injection. Anti-HBs antibody concentration above 10 IU/L is considered efficient. In cases with concentration below 10 IU/L like decreasing the concentration with time, immunosuppression, smoking, obesity, renal failure and liver disease a booster dose or a revaccination are suggested (1, 5).

## 3. Special Groups Needs Special Attention

The effectiveness of HBV vaccination and its response are studied in some particular diseases and conditions like patients with diabetes mellitus (DM), HIV, inflammatory bowel disease, chronic kidney disease, end stage renal disease requiring hemodialysis and the newborn infants (4, 7-10). However, we still lack data in some groups, as an instance, our data about is not still efficient about the effectiveness of vaccination during pregnancy on the prevalence of newborn infection and its side effects (11).

### 3.1. Diabetes Mellitus

Schillie et al. concluded that adult patients with DM have a reduced response to HBV vaccination and suggested an extended interval between the final doses of vaccine or using additional series of vaccination in this group of patients (8).

### 3.2. Patients With HIV Infection

HBV vaccination response is also reduced by HIV infection and some methods are available for improving this response like: using additional doses or adjuvants and intradermal injection. It is also believed that control of HIV replication and using of active antiviral therapy is of use for increasing the HBV vaccination response (10).

### 3.3. Patients Who Undergo Hemodialysis

Evidences show that patients who undergo HD cannot always produce enough antibodies in response to HBsAg following HBV vaccination; hence, infection with HBV remains a major health problem in these patients, with a high rate of morbidity and mortality (12-16). Although the first generation of HBV vaccine can protect these patients against the infection, it is less effective in them than in the normal population. In addition, it is reported that second generation of HBV vaccine has the same effect of the first generation in healthy people (1).

Some factors seem to be responsible for suboptimal response of HBV vaccination in patients who undergo HD; for example HLA-A1, B8, DR3 have been detected to have a higher prevalence in patients with lower vaccination responses (17). Female patients who undergo HD are more likely to be response to HBV vaccination than the male ones (17). Older age, malnutrition and infection with hepatitis C virus and human immunodeficiency virus can also have some effects on reduction of the effectiveness of HBV vaccination (18-21). Anemia is a common finding among patients who undergo HD which is usually corrected by human recombinant erythropoietin (EPO). In a series of 97 patients, resistance to EPO was associated with lower anti-HBs levels and seroconversion (22). It seems to be partly due to poor nutrition status and inflammation in these individuals (22). However, Fabrizi and his colleagues found that EPO injection has no effects on the HBV vaccination in patients who undergo HD (14).

It has been reported that several methods can improve the effect of HBV vaccination and its response in these patients. Fabrizi et al. showed that using of higher thymopentin doses as an adjuvant can lead to an increase in seroresponse rate of HBV vaccination (13). Some other adjuvants were also investigated. It seems that levamisole can also be effective for increasing the HBV vaccine response in the HD patients (15), but for a better decision more studies in this field are essential. HBV-AS04 containing adjuvant 3-O-desacyl-40-monophosphoryl lipid A which compatible with Engerix B Standard, makes an earlier and greater response to the HBV vaccination compared to the routine vaccination (1). Some studies have also evaluated the effect of intradermal and intramuscular injection of HBV vaccination in patients who undergo HD. It is said that in a short time follow up, intradermal route of HBV vaccination can lead to a stronger response than the intramuscular route; however, long term follow up did not show a meaningful difference between these two ways of injection (23).

## 4. The Present Situation Several Years After Starting the Successful HBV Vaccination Program

As mentioned above, it is well known that the HBV vaccination can lead to reduction of HBV prevalence and its complications, but now after many years of starting the HBV vaccination program there are still things to be taken care of. In a cross-sectional study by Al Ghamdi et al. evaluated the efficacy of primary HBV immunization in 238 medical students after 14-24 years (24). They found that their anti-HBs levels were meaningfully low (24). It has been said that with a worldwide immunization against HBV, some mutations like HBV's protein mutation (25) may occur in the HBV genome and this can be lead to higher levels of failure in making an appropriate response to the HBV vaccination. Chiara and coworkers showed that even a booster dose of HBV vaccination could not increase the anti-HBs antibody levels more than 10 IU/L in individuals with decreased response of HBV vaccination (anti-HBs antibody less than 2 IU/L) (26). In an study in Egypt It has also been reported that children with diabetics may not have effective concentrations of anti-HBs antibody after the primary vaccination (27). Moreover, there is a report of low long-term humoral immunity against HBV infection in vaccinated people in Bolivia (28). Patients with HIV infection may also lose their HBV vaccination response rapidly after completing the vaccination, so they need to be evaluated 6-12 months after finishing the primary vaccination series (29). Subjects with Down syndrome are another group that need a special follow up after HBV vaccination because of their low rate of seroconversion in response to the HBV vaccination (30). Additionally, patients with liver cirrhosis may also have a lower response to the HBV vaccination compared to the healthy population (31). Furthermore, many healthcare personnel may experience continuous lack of immunity to hepatitis B after completing vaccine series (32). Yildiz and colleagues evaluated the effect of HBV vaccine among children with steroid sensitive nephrotic syndrome and they observed a low response to the HBV vaccination and some relapses due to this vaccination in these cases. Finally, they suggested that the HBV vaccination should only be used in patients on low dose or withdrawal of steroid therapy (33).

Some studies suggested that using of a third generation vaccination in non- or low responder individuals to conventional vaccination can lead to making an effective level of anti-HBs antibody (more than 10 IU/l) (34); moreover, adding adjuvants like Advax to the current vaccines may be helpful for improvement of immunity level in low responder individuals (35). Also HEPLISAV, as a HBV vaccine, is suggested as a good option to be used among different groups such as healthcare personnel, patients with chronic kidney disease and healthy population. It can give an earlier seroprotection with fewer times and doses compared with Engerix-B as a second generation HBV vaccine (36, 37).

Despite the advances in HBV vaccine development and prevention therapy, we are now faced with following questions. When the booster dose and in which groups it must be administered? How exactly we should act with the time decreasing response to the HBV vaccination? Does HBV genome mutations should be considered for current HBV vaccination program? Is adding new adjuvants mentioned in databases, necessary? Which adjuvants and also which generations are better for each special group? To get a better understanding about these points, both original and secondary studies seem to be essential.
